# Comparison of tear Matrix Metalloproteinase 9 (MMP-9) estimation with Schirmer’s test in Ocular Surface Disorders

**DOI:** 10.22336/rjo.2024.42

**Published:** 2024

**Authors:** Sandeep Gupta, Sandeep Shankar, Sunandan Bhatta, Avinash Mishra, Ankita Singh

**Affiliations:** 1 Department of Ophthalmology, Military Hospital, Suratgarh, India,; 2 Department of Ophthalmology, Military Hospital, Gwalior, India,; 3 Department of Ophthalmology, Military Hospital, Agra, India,; 4 Department of Ophthalmology, Military Hospital, Jalandhar, India,; 5 Department of Ophthalmology, Military Hospital, Bathinda, India

**Keywords:** Ocular Surface Disorder, Schirmer’s test, Matrix Metalloproteinase 9, dry eye disease, OSD = Ocular Surface Disorder, MMP-9 = Matrix Metalloproteinase 9, DED = Dry eye disease, OSS = Ocular symptomatology Score, DEWS = Dry eye workshop

## Abstract

**Background:**

Ocular surface disorder (OSD) is a vexed eye problem and a diagnostic conundrum. Diagnosis has traditionally depended upon symptoms and tests like Schirmer’s, TBUT, staining with dyes, and tear meniscus height. Schirmer’s test is the most popular. However, the test strips irritate with reflex tearing - producing false high results. Matrix Metalloproteinase 9 (MMP) in the tear is believed to be expressed by stressed epithelial cells of the corneal surface - a key pathology in dry eye disease. This study attempts to compare the results of Schirmer’s test and MMP-9 so that the test can individually or severally add to a more definite diagnosis of dry eye disease.

**Materials and methods:**

100 eyes of 50 symptomatic patients underwent MMP-9 estimation and were divided into two groups (MMP-9+ve and MMP-9-ve). They were then sub-grouped as per DEWS-2007 based on Schirmer test levels and Ocular Symptomatology Score (OSS). The two groups were compared for severity of dry eye based on Schirmer’s test and OSS.

**Results:**

Mean Schirmer’s value was 12.85 (SD 7.07) for MMP-9+ve and 19.18 (SD 8.94) for MMP-9-ve patients. 80% of patients with severe dry eye and 55.6% of moderate dry eye patients were positive for MMP-9. 85% of the MMP-9 patients had OSS values of 2 or 3.

**Discussion:**

A higher OSDI and positive MMP-9 were shown to be correlated in a statistically remarkable way (p<0.001). The OSDI values of 0-12 for 3/44 (6.8%) positive results, 13-22 for 2/8 (25%) positive results, 23-32 for 4/14 (28.6%) positive results, and 33-100 for 13/35 (37.1%) positive results all showed an increase in MMP-9 positivity along with a rise in the subjective severity of the illness.

**Conclusion:**

MMP-9 compares well with Schirmer’s values and DED categories based on Schirmer’s. The result pointed towards the usefulness of this test in diagnosing patients who may have not yet manifested symptoms.

## Introduction

OSD (Ocular surface disorder) is a common disease that is multifactorial in origin and can cause severe eye discomfort, abundant watery eyes, and even serious visual impairment [1]. This term is used interchangeably with DED (dry eye disease). This is usually caused by either increased tear evaporation from the eye surface decreased aqueous tear production, or a combination of the two. Whatever the mechanism, stress related to the desiccation of the epithelium is almost invariably the cause of ocular surface inflammation. The resulting increased expression of inflammatory biomarkers is, thus, expected and observed. The Schirmer test has been the cornerstone of OSD [2] diagnosis for over a century. Its economy and simplicity have made it popular, but it still has certain drawbacks, including a lack of standardization and precision [3] and an irritating quality that can cause reflex tearing. Other tests, such as the evaluation of tear film breakup (TBUT) [4], although reproducible as well as less invasive than the Schirmer test, also have the disadvantage of instilling a local anesthetic, which can destabilize the tear film, causing a false improvement in TBUT. Staining methods also have limitations in that they cannot distinguish OSD from other causes of staining (e.g. medicinal) and are also less likely to be positive in mild or early OSD [4-6]. Tear osmolarity measurement is a new and promising tool in OSD diagnosis but has been shown to vary between samples, reflecting the inherent instability of this condition [7].

Recently, methods to measure tear MMP-9 levels have been developed. This is a hopeful breakthrough in the diagnosis of OSD. In conditions like OSD, stressed ocular surface epithelial cells produce proteolytic enzymes called MMPs. It has been discovered that increased levels of the MMP-9 biomarker correspond to DED and accurately represent the intensity of clinical symptoms in these instances [8]. In addition to relating to the severity of OSD, elevated levels of this biomarker can also be utilized to measure inflammatory lesions on the ocular surface. This is important information that was not previously disclosed by the Schirmer test. There is not much research contrasting the two approaches.

To bridge this knowledge gap, this study demonstrated that MMP-9 evaluation is a reliable substitute for the Schirmer test in OSD diagnosis and management. The hope is that the inflammatory markers in OSD will also result in the creation of new treatments that replace NSAIDs in the management of this prevalent and problematic illness.

## Materials and methods

### Study design

The pilot study was prospective and comparative and was conducted over three years in a tertiary care hospital.

### Inclusion criteria

All consecutive clinically symptomatic ocular surface disorders attending the OPD were incorporated into this study during the study period. Symptoms included foreign body sensation, dryness, excessive tearing, and eye irritation.

### Exclusion criteria

All patients with ocular allergy including VKC and papillitis, very severe dryness and poor ocular surface, and corneal pathology affecting tear film were excluded from the study, as the MMP-9 test could not be performed if the tear flow was low.

### Sample size calculation

A total of 100 eyes of 50 patients were evaluated as a pilot study.

### Blinding

Patients were recruited into the study by Observer 1, who collected histories and enrolled patients. Observer 2 administered the Schirmer test and MMP-9 assessment to all cases included in the study and started medication. Observer 3 evaluated all cases for visual acuity, repeated the Schirmer test and eye symptoms, and changed drugs as needed. All observers were blinded to other procedures.

### Procedure

**Fig. 1:**
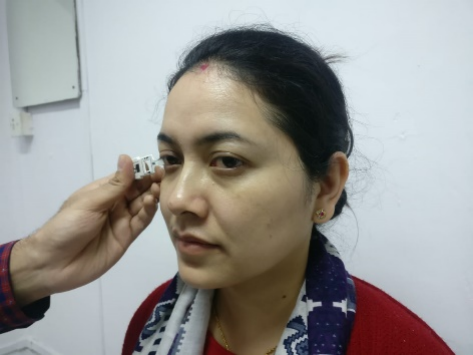
Sample collection by dabbing the “sampling fleece” on the patient’s conjunctiva

**Fig. 2:**
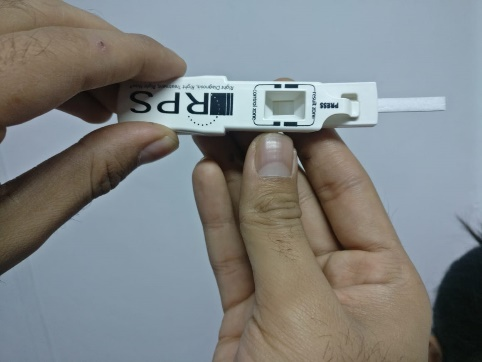
Assembling the test by gently placing the “Sampling Fleece” of the Sample Collector into the Sample Transfer Window of the Test Cassette Body

**Fig. 3:**
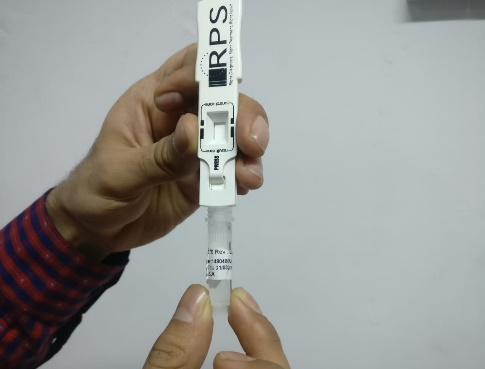
Immersion of the absorbent tip into the buffer vial

**Fig. 4:**
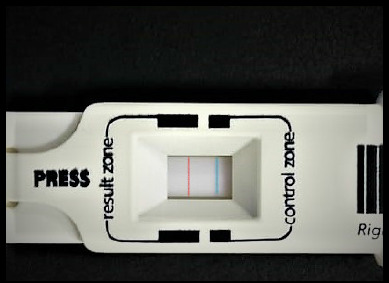
Reading is taken after 10 min. A positive result with a blue line in the control zone and a red line in the result zone

All enrolled patients underwent the Schirmer test, MMP-9 assessment, and OSS assessment. Commercially available Schirmer strips were used to conduct the Schirmer test. Both eyes had been evaluated simultaneously. The strips were folded 5 mm from the edges and placed on the lower bow at the intersection of the inner 2/3 and the outer 1/3 of the cover. After five minutes, readings were recorded on labeled, moistened paper with the patients instructed to keep their eyes open in an environmentally protected room. The MMP-9 test was performed with the Inflamma Dry kits, where tears were collected with a sponge provided in the kit, which was afterward added to the kit and whether the buffer solution contained elevated MMP-9 levels or not was identified by the line lines (**Fig. 1-4**).

Depending on whether their MMP-9 results were positive (Group I) or negative (Group II), the patients were divided into two groups.

Moreover, subgroups had been created using Schirmer’s test levels from DEWS-2007, as well as these were assessed at the beginning of the study as follows:

Mild dry eye - Schirmer’s value 10 to 15; Moderate dry eye - Schirmer’s value 5 to10; Severe dry eye - Schirmer’s value 2 to 5.

Patients with Schirmer’s value < 2 (very severe disease) were not included in the study as it would not have been possible to perform the MMP-9 test with near-absent tears.

Every case was assessed using the ocular symptomatology score (OSS), which has a 0 to 4 range. For this investigation, OSS was defined as:

0- No symptoms;
1- Minimal symptoms, duration < 4 hrs. daily;
2- Moderate symptoms, duration > 4 hrs. daily;
3- Severe symptoms relieved by medications;
4- Severe symptoms affecting daily activities.

## Results

In all, one hundred eyes had been used in the investigation. MMP-9 test had been positive in 40 eyes. The patients who took part in the study were 60.26 years old on average. The mean age of the patients in group I (MMP-9-positive) was 58.05, while the mean age of the group II (MMP-9-negative) patients was 61.73. Their income disparities were not substantial. In all, 58 women and 42 men participated in the investigation. Eighteen patients were male and twenty-two were female among the forty who tested positive for MMP-9.

a) Relation between MMP-9 and Schirmer’s values

**Table 1** shows Schirmer scores for MMP-9-positive patients averaged 12.85 with a standard deviation of 7.07, while those for MMP-9-negative patients averaged 19.18 with a standard deviation (SD) of 8.941. With an unpaired t-test p-value less than 0.001, the mean difference was 6.33, thus being significant. As a result, MMP-9-positive patients had much lower Schirmer scores.

**Table 1:** Mean Schirmer values in Group I and II

		Number of eyes	Mean Sch. value	Std. Deviation	Std. Error Mean
	MMP-9 +	40	12.85	7.069	1.118
	MMP-9 -	60	19.18	8.941	1.154

b) Relation of MMP-9 with Schirmer’s categories

**Table 2** shows that, based on the Schirmer classification, MMP-9 was positive in four out of five patients (80%) with a severe dry eye. In contrast, patients with moderate dry eye accounted for 55.6% of the positive MMP-9 tests, while only 22.5% of individuals with normal tear secretion achieved this status. The Chi-Square test found a substantial (p<0.05) correlation between MMP-9 positivity and the severity of Schirmer grade.

**Table 2:** Schirmer’s Category and MMP-9 Cross tabulation

				MMP-9		Total
				Negative	Positive	
	Schirmer’s Category
		Normal	Count	31	9	40
			% in Schirmer’s Category	77.5%	22.5%	100.0%
		Mild	Count	16	12	28
			% in Schirmer’s Category	57.1%	42.9%	100.0%
		Moderate	Count	12	15	27
			% in Schirmer’s Category	44.4%	55.6%	100.0%
		Severe	Count	1	4	5
			% in Schirmer’s Category	20.0%	80.0%	100.0%
	Count	60	40	100
Total						
			% in MMP Group	100.0%	100.0%	100.0%

**Key (for the study)**

**Table:** 

*Sch. Value Category*	*Category*
*>15 Normal*	*Normal*
*15-10 Mild*	*Mild*
*5-<10 Moderate*	*Moderate*
*2-<5 Severe*	*Severe*
*<2 Not considered*	*Not considered*

As a result, according to Schirmer’s test, a higher percentage of MMP-9-positive patients had dry eye symptoms that were more severe.

c) Relation of OSS with Schirmer’s categories

**Table 3** shows that nineteen out of forty eyes (47.5%) had an OSS score of two on a normal Schirmer test. On the other hand, 4 out of 5 eyes (80%) had a severe Schirmer test, and 10 of 27 eyes (37%) had a moderate Schirmer test. Three was the OSS. A moderate Schirmer test was also performed on all four eyes (100%) with an OSS score of 4. The Chi-Square test revealed a significant positive correlation between the OSS grade on Schirmer’s test and the severity of DED.

**Table 3:** Schirmer’s Category and OSS Cross tabulation

					OSS			Total
				1	2	3	4	
	Schirmer’s Category	Normal	Count	6	19	15	0	40
			% in Schirmer’s Category	15.0%	47.5%	37.5%	0.0%	100.0%
			% within OSS	42.9%	39.6%	44.1%	0.0%	40.0%
			% of Total	6.0%	19.0%	15.0%	0.0%	40.0%
		Mild	Count	1	22	5	0	28
			% in Schirmer’s Category	3.6%	78.6%	17.9%	0.0%	100.0%
			% within OSS	7.1%	45.8%	14.7%	0.0%	28.0%
			% of Total	1.0%	22.0%	5.0%	0.0%	28.0%
		Moderate	Count	6	7	10	4	27
			% in Schirmer’s Category	22.2%	25.9%	37.0%	14.8%	100.0%
			% within OSS	42.9%	14.6%	29.4%	100.0%	27.0%
			% of Total	6.0%	7.0%	10.0%	4.0%	27.0%
		Severe	Count	1	0	4	0	5
			% in Schirmer’s Category	20.0%	0.0%	80.0%	0.0%	100.0%
			% within OSS	7.1%	0.0%	11.8%	0.0%	5.0%
			% of Total	1.0%	0.0%	4.0%	0.0%	5.0%
			Count	14	48	34	4	100
			% in Schirmer’s Category	14.0%	48.0%	34.0%	4.0%	100.0%
Total			% within Pre_OSS	100.0%	100.0%	100.0%	100.0%	100.0%
			% of Total	14.0%	48.0%	34.0%	4.0%	100.0%

***Key:*** Ocular symptomatology score (for the purpose of the study, adapted from Ocular Surface Disease Index) has been defined as

**Table:** 

*0-*	*No symptoms*
*1-*	*Minimal symptoms, duration less than 4 hrs. daily*
*2-*	*Moderate symptoms, duration more than 6 hrs. daily*
*3-*	*Severe symptoms relieved by medications*
*4-*	*Severe symptoms affecting daily activities*

d) Relation of OSS with MMP-9 positivity

**Table 4** shows that only two patients (5%) out of the 40 with positive MMP-9 had an OSS of 4, while 18 patients (45%) and 16 patients (40%) had scores of 3, 2, and 4 patients, respectively, had scores of 2. MMP-9 patients with a score of 4 (10%) had an OSS value of 1.85%, indicating moderate results. MMP-9 and OSS (chi-square test: p>0.05) positivity showed no clear correlation.

**Table 4:** MMP-9 and OSS Cross tabulation

				OSS			Total
			1	2	3	4	
	Negative	Count	10	32	16	2	60
		% within MMP-9 category	16.7%	53.3%	26.7%	3.3%	100.0%
		% within the OSS category	71.4%	66.7%	47.1%	50.0%	60.0%
MMP-9		% of Total	10.0%	32.0%	16.0%	2.0%	60.0%
	Positive	Count	4	16	18	2	40
		% within MMP-9 category	10.0%	40.0%	45.0%	5.0%	100.0%
		% within the OSS category	28.6%	33.3%	52.9%	50.0%	40.0%
		% of Total	4.0%	16.0%	18.0%	2.0%	40.0%
		Count	14	48	34	4	100
		% within MMP-9 category	14.0%	48.0%	34.0%	4.0%	100.0%
Total		% within the OSS category	100.0%	100.0%	100.0%	100.0%	100.0%
		% of Total	14.0%	48.0%	34.0%	4.0%	100.0%

## Discussion

Keratoconjunctivitis sicca (KCS), OSD, or just “dry eye” are other names for DED, which has proven to be a challenging clinical condition to define. It was originally thought to be simply a disorder of decreased tear flow, but it was later realized that DED disrupts the normal balance between production and evaporation. Even later, ocular surface inflammation presence was identified as an important factor. The most accepted definition of DED by the DEWS (Dry Eye Workshop) [1] considers all of the above. It emphasized the presence of symptoms associated with DED, including discomfort as well as visual disturbances, and the damage it causes to the eye surface. Elevated osmolarity in the tear film has also been identified as a prominent pathology that induces inflammation of the tissue forming the ocular surface. Dry eye cannot be diagnosed based on symptoms alone, as many different tear film disorders and ocular surface pathologies can cause symptoms similar to dry eye. In addition, the environmental conditions that the patient encounters daily influence the development and variation of symptoms and signs. In contrast, less than 57% of dry eye patients experience symptoms 2-5. This observation could be explained by the distinct etiology and pathophysiology of dry eye or by the fact that symptoms frequently appear before other symptoms [6].

The Schirmer tear test employment for over a century has been described as the most widely used diagnostic procedure for dry eye [7]. However, the test lacks standardization [8] and has inaccuracy and non-reproducibility errors, because the test strips on the eye surface irritate and cause reflex lacrimation [9]. In addition, the test is limited in its ability to quantify tear flow [10], while factors affecting dry eye evaporation are not considered at all [8]. Other factors limiting the application of the Schirmer tear test as a device for diagnosis are time inefficiency, patient discomfort, and lack of sensitivity. However, the cost savings achieved in inexpensive test strips and the relative ease of use of the test have helped the Schirmer test, which is the most widely applied, be commonly used to assess the excretory portion of dry eye. TBUT (Tear break time) [11-13] is another test that is quite widely used to diagnose DED. This involves using fluorescein dye to stain the eye surface to look for dry patches. Since TBUT is repeatable and minimally invasive, it is more dependable than the Schirmer test. However, the local anesthetic must be instilled before the procedure is thought to destabilize the tear film, resulting in a falsely low TBUT [11-13]. In addition, unlike the Schirmer test, this test does not provide direct information about tear secretion. DED has also been diagnosed using patterns from ocular surface staining. One of them is to use dyes such as fluorescein, and Lissamine Green, with Rose Bengal. The staining pattern can then be compared to standard photographs, and classified according to the severity of DED [10,14,15]. Ocular surface staining’s drawback is that it cannot clinically distinguish dry eye from other conditions, such as poor eyelid attachment, drug toxicity (including local anesthesia), trauma, or infection, which also cause staining [16]. Additionally, these techniques become uncomfortable and ineffective in mild or early dry eye [16].

In primary care settings, other than general clinical practice research, ophthalmic technicians treat most patients with dry eyes as part of their therapy. Typically, patients report when their symptoms started. Patient mobility is restricted by the ability of support personnel to accurately perform TBUT or else corneal staining before an ophthalmologist evaluates the patient clinically. Based on the above, it was concluded that DED should be recognized and diagnosed based on symptoms that eye technicians can provide. This realization led to the progression of the OSDI (Ocular Surface Disease Index) [17]. The patient answers 12 questions as part of the OSDI, each being graded on a subjective basis. The test is usually performed during the first presentation and then at a specified time during follow-up. Thus, it helps both in diagnosis and in evaluating the therapeutic benefit of drugs. Although the approach is effective, it is a purely subjective assessment that lacks specificity and is vulnerable to analytical errors dependent on the user, making it unsuitable for regular use in clinical practice [1,18,19]. Inherent shortcomings of symptom-based diagnostic scales or routine diagnostic tests a simple but reliable test is needed to avoid low specificity and standardization. Evaluation of MMP-9 (matrix metalloproteinase-9) is a step in that direction. MMP-9 is a member of the matrix metalloproteinases family. These are proteolytic enzymes secreted by cells lining the surface epithelium of the eye, especially when exposed to stress such as drought. According to one study [20], their normal level in tears varies between 3 and 40 ng/ml. It has been proposed that alterations in the corneal epithelium’s barrier function cause eye irritation experienced by DED patients. It is mainly related to an inflammatory process. Matrix metalloproteinases, or MMP-9, are thought to be a marker of inflammation. Studies have shown that their levels are consistently developed in the tears of patients with DED [21]. It seems that MMP-9 is involved in the physiological process that leads to corneal epithelial desquamation. Measuring MMP-9 does not provide information on tear production or formation. However, increased corneal epithelial desquamation in dry eye is likely associated with a decreased corneal epithelial barrier function, and a surface irregularity of the cornea [20]. Other ocular surface pathologies in which MMP-9 activity is elevated include diseases like Sjogren’s syndrome and blepharitis. However, it should be remembered that these conditions finally manifest themselves clinically with the development process of inflammatory dry eye disease and that MMP-9 evaluation with a qualitative test would help in accurately identifying such conditions [21]. Other eye diseases that have been found to cause elevated MMP-9 levels include infection [22], allergy [23], pterygium [24], and conjunctival chalazion [25]. However, these conditions can be relatively easily distinguished from DED because of the associated unique signs and symptoms. Thus, detection of elevated MMP-9 levels can help diagnose DED early, when it is less severe and more treatable. However, measuring MMP-9 in tears in the past required tools for sophisticated laboratory techniques, which prevented its widespread adoption. A test module that is a point-of-care immunoassay similar to fashion tests (e.g. beta-hCG) may be an option to achieve wider acceptance of the test. “InflammaDry” is one such MMP-9 immunoassay. It is a single-use, non-invasive, bedside test, reasonably simple to employ. Simbirsk et al. [26] published a study on its sensitivity and specificity. For clinically diagnosed DED [26], a multicenter study with 143 cases and 63 controls revealed a sensitivity of 85% and a specificity of 94%. The test presented positive (97%) and negative (73%) predictive values. The test was performed similarly in another study. It presented 81% positive and 98% negative cases, compared to a clinical assessment of dry eye [27]. Clinically diagnosed cases of DED were included in this study.

All patients receiving medications that affected the eye surface or systemic disorders were excluded. Forty of the one hundred eyes that were part of the study, had positive results from the MMP-9 test. This was similar to the Messmer et al. study [28], in which the inflammation kit produced positive results in 22 eyes (21.8%) out of 101 subjects tested for tear film MMP-9. In three out of the 54 control patients (5.6%) and 19 out of the 47 dry eye patients (40.4%), the MMP-9 level in the tear film increased to 40 ng/mL or higher.

A higher OSDI and positive MMP-9 were shown to be correlated in a statistically remarkable way (p<0.001). The OSDI values of 0-12 for 3/44 (6.8%) positive results, 13-22 for 2/8 (25%) positive results, 23-32 for 4/14 (28.6%) positive results, and 33-100 for 13/35 (37.1%) positive results all showed an increase in MMP-9 positivity along with a rise in the subjective severity of the illness.

## Conclusion

The study showed that assessing MMP-9 using qualitative assays can be a viable alternative to traditional tests, namely Schirmer and TBUT. It was shown that it was independent of the technician/observer and did not destabilize the tear film, at the same time being easy to dose and providing valuable information about the inflammatory component of the disease. The Schirmer test tear film evaluation and MMP-9 positivity were found to be related to the study, although an association between OSS and MMP-9 could not be determined. The research findings provided a good case for adding this test method to the dry eye diagnostic test case, although more studies are needed to prove the cost-benefit ratio.
